# Robots and AI are not one moral category: why the distinction matters for ethical and conscious systems

**DOI:** 10.3389/frobt.2026.1776097

**Published:** 2026-02-20

**Authors:** Ahmet Küçükuncular

**Affiliations:** Near East University, Faculty of Economics and Administrative Sciences, Nicosia, Cyprus

**Keywords:** AI ethics, artificial consciousness, embodied artificial intelligence, meaningful human control, moral agency, moral appearance, robot ethics, sociotechnical systems

## Introduction

Calls that pair ethical and conscious AI with ethical and conscious robots may feel natural. Many contemporary robots use machine learning, and many AI systems are described in agentive terms. Yet the pairing can hide a conceptual shortcut. It quietly suggests that AI ethics and robot ethics are the same moral question applied to different shells. My claim in this opinion piece is modest but consequential: treating robotics and AI as a single moral category encourages avoidable category mistakes about where moral agency sits, where harms arise, how responsibility is attributed, and what consciousness claims could plausibly mean in deployed systems.

The overlap is real but not identity. Robotics is best understood as the engineering of embodied artefacts that sense and act in the physical world. AI is best understood as a family of computational techniques that can be embedded in many artefacts, including robots, but also in disembodied services such as decision support tools, recommender systems, and conversational agents (Riesen, 2025). The distinction defended here is not offered as a new ethical theory. It functions as a scoping rule for interdisciplinary work. It helps prevent recurring errors in evaluation, especially the tendency to import the ethical agenda of disembodied algorithmic systems into contexts where physical presence and bodily interaction are decisive, or to import debates about the moral status of social robots into contexts where there is no body, no situated action, and no human robot relationship (Moon et al., 2021; Torras, 2024).

This matters for research on ethical and conscious systems. Ethical performance is not only about internal decision rules; it is also about pathways of influence, constraint, and harm in real settings (Mittelstadt et al., 2016; Santoni De Sio and Van Den Hoven, 2018). Consciousness claims, if they ever become technically serious, will still intersect with embodiment, user perception, and accountability in ways that differ sharply between robots and software agents (Dehaene et al., 2017; Gray et al., 2007). Accordingly, I proceed in three steps. First, I separate overlap from equivalence by distinguishing computational cores from embodied systems. Second, I show why embodiment changes ethical evaluation by altering harm profiles, moral appearance, and responsibility pathways. Third, I translate this distinction into a practical discipline for research communication, so that authors, reviewers, and governance oriented readers can assess claims about ethical and conscious systems without conflating the relevant object of evaluation.

### Overlap without equivalence

A useful starting point is to separate the computational core from the embodied system. A robot may include AI modules, but it also includes sensors, actuators, safety interlocks, mechanical design, and a deployment environment. Conversely, many AI systems have no body at all, yet still shape behaviour through information, ranking, and gatekeeping (Mittelstadt et al., 2016). The ethical object is therefore rarely the AI model or the robot platform in isolation. It is the sociotechnical arrangement as a whole, including design choices, organisational incentives, user practices, and regulation (Riesen, 2025; Vallor and Vierkant, 2024). This point is familiar within sociotechnical and responsible robotics approaches, but my present emphasis is that the presence or absence of embodiment is not a minor implementation detail. It alters which ethical questions are primary, and which evidence would be relevant when assessing agency and consciousness claims.

Philosophical work on artificial agency helps clarify why the boundary matters. Floridi and Sanders (2004) argue that we can evaluate artificial agents at different levels of abstraction, without assuming that the artefact is a humanlike moral agent. That lens is valuable, but the choice of level is not free of engineering reality. Embodiment expands a system’s causal footprint into the physical domain. A robot can collide, restrain, touch, obstruct, or physically shepherd. Even when its intelligence is modest, its body can create ethical stakes that look closer to product safety, bodily autonomy, and coercion than to statistical bias in classification (Moon et al., 2021; Torras, 2024). In contrast, disembodied AI can generate ethically significant effects without any physical presence, through epistemic authority, persuasive interaction, ranking power, or institutional gatekeeping. Treating these cases as if they raised the same ethical problems as embodied robots risks misspecifying both harms and responsibilities, and it encourages over general claims about agency or consciousness that are not supported by the relevant interaction context (Mittelstadt et al., 2016).

This motivates a discipline for research claims. When one asks whether a system is ethical, one ought to specify which of at least three targets one means: (1) ethical reasoning competence, meaning the quality of internal deliberation or value alignment (Moor, 2006), (2) ethical behaviour in context, meaning the observed effects of actions and interactions in a setting (Moon et al., 2021), (3) ethical governance, meaning how responsibility, oversight, and accountability are structured around the system (Santoni De Sio and Van Den Hoven, 2018). The proposed AI versus robot distinction sharpens this discipline by forcing an explicit answer to a prior question: what is the system under evaluation, a disembodied computational service, an embodied artefact, or a wider sociotechnical arrangement in which embodiment plays a constitutive role. Without this clarification, ethical appraisal can slide between targets and levels of abstraction, producing apparent disagreement that is in fact a mismatch of evaluative objects.

Machine ethics has long noted that systems can be ethical impact agents without being full moral agents (Floridi and Sanders, 2004; Moor, 2006). Robots, by being physically active and often socially present, tend to become ethical impact agents by default, even before we settle questions about moral agency. Disembodied AI systems can also become ethical impact agents by default, but typically through different pathways, such as differential access to opportunities, behavioural steering, or delegations of authority within organisations. Recognising overlap without equivalence makes these pathways easier to separate analytically, and it clarifies why collapsing AI ethics and robot ethics can create different category mistakes in different contexts.

### Different harm profiles

Much of the modern ethical AI agenda grew around algorithmic mediation: fairness, accountability, transparency, privacy, and downstream social impacts of automated decisions (see, for example, Jobin et al., 2019; Mittelstadt et al., 2016). These concerns remain relevant when AI is embedded in robots. A care robot that allocates attention, flags risk, or prioritises tasks can reproduce bias just as a disembodied triage system can (Sharkey and Sharkey, 2012). Yet robot ethics adds dimensions that are easy to miss when everything sits in one basket. This section therefore isolates what embodiment changes in ethical evaluation, and why that change should alter research design, evidence standards, and governance expectations.

First is kinetic risk and bodily autonomy. The moral difference between a classifier and a mobile robot is not merely that one moves. Movement changes the kinds of harm that are salient, the time horizons of safety, and the evidential standards we should demand. Failure in a recommender system is often informational or distributive. Failure in an embodied robot can be immediate and bodily. This shifts ethical evaluation towards verification, fail safe design, and forms of human control that support timely intervention (Santoni De Sio and Van Den Hoven, 2018; Verhagen et al., 2024). This is also a methodological point: what counts as adequate assurance differs by domain. In disembodied AI, evaluation often prioritises representativeness, error disparities, contestability, and *post hoc* explanation. In robotics, assurance must additionally address mechanical reliability, hazard analysis, safe stopping, and the conditions under which human override is practically possible, not merely nominal.

Second is corporeal social influence. Robots have physical presence, can occupy space, can touch, and can create a sense of copresence. That changes what manipulation, consent, and vulnerability look like. Importantly, some of these concerns arise regardless of how advanced the underlying AI is (Moon et al., 2021). The ethical difficulty can be driven by embodiment itself, not by the sophistication of the model. A useful contrast is that disembodied AI frequently influences through informational pathways, such as ranking, recommendation, nudging, or institutional gatekeeping, whereas robots can additionally influence through spatial positioning, proximity, and touch, which can render consent more ambiguous and refusal more difficult in practice.

A practical way to keep both tracks visible is to separate harms into two spaces: (a) informational and institutional harms, such as bias, opacity, privacy loss, unequal access, and power asymmetries (Jobin et al., 2019; Mittelstadt et al., 2016), and (b) kinetic and relational harms, such as bodily safety, unwanted touch, spatial coercion, dependency, deception through social cues, and erosion of skills or relationships (Moon et al., 2021; Sharkey and Sharkey, 2012; Torras, 2024). The analytic value of this separation is not taxonomic elegance but governance clarity: it helps specify which harms are plausible in a given deployment and which forms of evidence, testing, and oversight are proportionate.

The second space is where category mistakes become costly. If we treat robotics as applied AI, we may overweigh what is easiest to measure in software and under weigh what is hardest but decisive in embodied interaction. For interdisciplinary audiences, this is the key practical implication of insisting on the distinction: it realigns what reviewers and policy assessors should ask for. A robot may satisfy common AI ethics expectations while still being ethically unacceptable due to interaction level risk, and a disembodied system may satisfy safety-oriented criteria while remaining ethically unacceptable due to institutional harms. Treating these as one evaluative basket blurs that difference and weakens accountability.

A related issue is norm compliance. There is growing interest in robots that learn and enact social norms. But norms are not automatically ethical, and encoding them can amplify bias, paternalism, and politically entrenched expectations. Recent critique catalogues multiple ways norm compliant robots can reinforce problematic norms and induce harmful norm change (Coggins and Steinert, 2023). That critique lands differently in robotics than in disembodied AI, because robots enact norms through physical presence and behaviour that users experience as interpersonal. This again illustrates why embodiment changes the moral interface: the same norm encoded in software may be experienced as bureaucratic exclusion, while enacted by a robot it may be experienced as interpersonal correction, pressure, or even coercion.

### Moral status and moral appearance

The pairing of conscious AI and conscious robots also raises a sharper philosophical question. Are these morally the same claim? If conscious means phenomenal consciousness, meaning there is subjective experience, then moral patiency plausibly depends on experience rather than on a chassis. A conscious disembodied system would be a moral patient in the same broad sense as a conscious robot, even if it lacked a body. That is why scientific discussions emphasise the need to clarify what functions and architectures would count as evidence for consciousness claims, rather than relying on surface behaviour (Dehaene et al., 2017). This paper therefore distinguishes two separable issues that are often conflated when AI and robots are discussed together: the metaphysical question of whether a system is conscious, and the practical question of how consciousness like claims will be interpreted and operationalised in real deployments.

Robots, however, introduce a second phenomenon that cannot be ignored: moral appearance. Humans infer mind from cues, and these inferences shape moral judgement. Mind perception research suggests people organise these inferences along dimensions such as experience and agency, and these perceptions predict moral responses (Gray et al., 2007). In human robot interaction, anthropomorphism and perceived intelligence are measurable constructs that affect trust, likeability, and perceived safety (Bartneck et al., 2009). Recent work also operationalises perceived moral patiency of social robots, showing that people can attribute morally relevant vulnerability to robots in systematic ways (Banks and Bowman, 2023). Disembodied AI can also generate moral appearance, particularly through linguistic fluency, conversational framing, and the presentation of confident outputs, but the cues are narrower and the interaction is typically mediated through screens and institutional workflows rather than co present behaviour. The distinction matters because the evidential basis for mind attribution, and the channels through which users become vulnerable to manipulation or deference, differ across these contexts.

This creates an asymmetry that is ethically important even before we resolve the metaphysical and the ontological. A robot can be treated as if it were conscious because embodiment supplies social cues such as gaze, rhythm, proximity, and touch. Coeckelbergh (2010a), Coeckelbergh (2010b) argues that moral consideration can be shaped by social relations and moral appearances, not only by hidden mental properties. At the same time, the robot rights debate has prompted warnings about mistaking human projections for genuine moral status, and about the political and legal implications of granting rights language to artefacts (Birhane et al., 2024). A parallel warning applies to disembodied AI, where agency like language and consciousness rhetoric can encourage misplaced deference to outputs, over trust in system competence, or the diffusion of responsibility within organisations. The category mistake differs, but the governance risk remains; moralised narratives can substitute for clear accountability.

Empirically, the consequences are no longer speculative. If people judge violence against robots as morally charged, that changes how we should think about deployment, user training, and acceptable design affordances. Recent experimental work suggests that people’s moral judgements about harming robots can be measured and meaningfully vary with context (Archer et al., 2025). Related studies show that both anthropomorphising and dehumanising tendencies can shape moral and social responses to robots, which matters for accountability and user protection (Wieringa et al., 2025). These findings strengthen the practical case for separating moral status from moral treatment: even if no credible evidence for robot consciousness exists, predictable human responses generate ethically relevant duties regarding design, disclosure, and the prevention of manipulation and dependency.

So the moral landscape has at least two layers: (i) moral status claims, meaning whether there is consciousness and therefore potential moral patiency (Dehaene et al., 2017), and (ii) moral treatment dynamics, meaning how humans will treat the system as minded, and what duties arise from predictable human responses, including risks of attachment, deference, and manipulation (Banks and Bowman, 2023; Coeckelbergh, 2010a; Wieringa et al., 2025). The key point is that these layers invite different evidential standards. Moral status claims require unusually stringent justification. Moral treatment dynamics can be assessed empirically through interaction studies and deployment evidence, without presupposing consciousness.

Robots intensify the second layer. Disembodied AI can also elicit social responses, but embodiment amplifies and diversifies the channels of influence. This is why ethical and conscious robots are not simply ethical and conscious AI with a body. The body is part of the moral interface. The practical consequence is that ethical appraisal should not treat “consciousness like” impressions as interchangeable across domains. A robot’s embodied cues can generate moral appearance that demands design and governance responses even in the absence of consciousness, whereas disembodied AI more often generates moral appearance through epistemic authority and linguistic performance, demanding different safeguards, disclosure norms, and accountability structures.

### Agency and responsibility in embodied systems

Ethical systems research often slides between two senses of agency: causal agency, meaning the system makes things happen, and moral agency, meaning the system can be held responsible in a normative sense (Floridi and Sanders, 2004; Vallor and Vierkant, 2024). Taxonomies of machine ethics make room for machines that have ethical impact without being full ethical agents (Moor, 2006). Robots strain responsibility practices because their behaviour is situated, adaptive, and sometimes learned, creating well known responsibility gaps when outcomes are not reasonably foreseeable by designers or operators (Matthias, 2004). This section clarifies why embodiment makes that slide more consequential, and why responsibility attribution cannot be repaired by treating ethics as an internal module alone.

Adding an ethical reasoning module does not close this gap. A robot can deliberate well and still be embedded in a pipeline of incentives, training regimes, user pressures, and physical constraints that distribute control. Frameworks for meaningful human control aim to preserve accountability through design requirements that connect human reasons, oversight, and system behaviour (Santoni De Sio and Van Den Hoven, 2018). More recent conceptual work shows that meaningful human control is not a single simple requirement but a family of interpretations that shift across domains and governance goals, making operationalisation and measurement central research problems (Robbins, 2024; Verhagen et al., 2024). This is precisely where the AI versus robot distinction yields practical leverage. Disembodied systems often distribute responsibility through institutions and data pipelines, whereas embodied robots additionally distribute it through physical coupling, real time constraints, and interaction dynamics that can make oversight fragile. Treating these cases as equivalent invites either over attribution of responsibility to the artefact, or under specification of the human and organisational conditions required for accountable deployment.

Embodied cognition perspectives underline why this mapping is not optional. If cognition is tightly coupled to action and environment, then ethical behaviour will also be tightly coupled to environment (Wilson, 2002). The extended mind tradition likewise emphasises that cognitive processes can be distributed across agent and world, which is a useful warning against locating ethical competence in an internal module alone (Clark and Chalmers, 1998). In robots, this coupling is literal. Treating robotics as merely applied AI risks under specifying the physical and organisational conditions under which ethical behaviour can be expected. The point is not to deny sociotechnical continuity, but to ensure that evaluation and governance track the full control loop: sensors, actuation, bodies, spaces, and organisational incentives. Without that, responsibility gap discussions risk becoming abstract, while the most decisive sources of harm and accountability failure remain located in deployment conditions rather than in models.

## Discussion: keeping the baskets distinct without splitting the field

Separating robotics and AI conceptually may not require separating communities or journals. It does, however, require resisting a hidden equivalence. For research on ethical and conscious systems, I propose a simple reporting norm that would reduce conceptual slippage while still encouraging integrated work. Every contribution should state, explicitly, what its target of ethical analysis is and where embodiment enters the story (Riesen, 2025; Torras, 2024).

Concretely, authors could answer four questions at the outset, so that subsequent claims about ethics, consciousness, and responsibility remain anchored to stable evaluative objects.

- What is the system boundary: model, robot platform, or sociotechnical deployment (Riesen, 2025).
- What is the embodiment level: disembodied service, screen based agent, mobile robot, or physically interactive robot (Moon et al., 2021).
- What is the ethical target: reasoning competence, behaviour in context, or governance and accountability (Moor, 2006; Santoni De Sio & Van Den Hoven, 2018).
- What is the consciousness claim, if any: access consciousness, phenomenal consciousness, or human perceived mindedness (Dehaene et al., 2017; Gray et al., 2007).

This norm is intentionally lightweight. It does not demand that every paper cover every target. It asks only that authors specify what they are and are not claiming, and which kinds of evidence would be relevant given the system boundary and embodiment level. At the same time, this norm is not bureaucratic; it is a way of aligning methods with moral risk. For instance, introspective self control mechanisms evaluated only through decision traces can miss their primary ethical function in robots, namely, regulating physical action under uncertainty and under constraint (Santoni De Sio and Van Den Hoven, 2018; Verhagen et al., 2024). Similarly, work on visual self perception can mean very different things in an embodied robot, where self perception supports safe action and bodily boundaries, versus in disembodied AI, where self modelling may primarily concern epistemic confidence and decision calibration (Riesen, 2025; Wilson, 2002). In review terms, the four questions provide a simple checklist: are the evaluation methods appropriate to the system’s dominant risk space, and are the governance implications proportional to the kind of harm and responsibility distribution that is plausible in deployment. In policy terms, the same clarifications reduce the temptation to generalise from a narrow benchmark or laboratory study to broad claims about ethical alignment or consciousness.

To the motivating question, are ethical and conscious AI and ethical and conscious robots morally the same? Philosophy suggests a split answer. Moral patiency, if grounded in conscious experience, is in principle independent of embodiment (Dehaene et al., 2017). Moral practice, responsibility, and influence are deeply shaped by embodiment and social relation, including moral appearances and predictable human projections (Coeckelbergh, 2010a; Gray et al., 2007; Moon et al., 2021). Treating them as the same question risks building consciousness sounding narratives for systems whose primary ethical risks lie in bodies and settings, while also overlooking that disembodied systems could, in principle, raise serious moral status questions if consciousness ever becomes a defensible empirical claim. The distinction herein defended therefore does not split the field. It makes the field easier to govern, by ensuring that ethical evaluation and consciousness talk remain accountable to the specific interaction context, harm profile, and responsibility pathway at stake.

Keeping two baskets in view is not pedantry. It is a condition for building systems that are ethically aligned and ethically governable. A research topic that jointly considers ethical and conscious AI and robots is valuable. My suggestion here is simply that the joint framing should be accompanied by a minimal discipline of specification, so that interdisciplinary work avoids category mistakes, and so that disagreements can be traced to genuine normative differences rather than to shifting system boundaries, embodiment levels, or targets of ethical appraisal.
